# Appendicitis

**Published:** 1902-02

**Authors:** 


### Appendicitis.

A recent number of the *New York Medical Journal* contains a paper on appendicitis by Dr. John B. Deaver, in which the author states that although he operates upon several hundred cases annually, almost every case teaches him some new lesson in the living pathology of the disease. He is convinced that the only true conservatism in the treatment of appendicitis lies in recourse to the scalpel as soon as the diagnosis has been established.

Everyone who wishes to become familiar with appendicitis must first familiarize himself with its ante-mortem pathology. An early diagnosis is of the utmost importance, since every death except those occurring from the fulminating types could have been prevented by use of the knife at the proper time.

The three cardinal symptoms are: (1) *Pain,* which shortly becomes localized over either the base of the appendix, McBurney's point, or its tip, which may be anywhere in the abdominal cavity. (2) *Tenderness,* which may be general or local. General abdominal pain with tenderness localized over McBurney's point is of great diagnostic value. The greatest tenderness is usually localized over a small area, and is best elicited by pressure of a single finger. (3) *Rigidity* of the abdominal muscles, which can often be determined only by the gentlest possible touch. The rigidity is sometimes so marked that it gives the idea that a mass is present.

The diseases hardest to distinguish from appendicitis are typhoid fever, extra-uterine pregnancy, cholecystitis, and acute mechanical obstruction of the bowel.

The practice of waiting to decide whether a case of appendicitis be one suitable for operation or not is to be absolutely condemned. The only possible excuse for delay is, the writer thinks, uncertainty as to the diagnosis.

The ideal time for operation is during the stage of appendicular colic, before inflammation is well established. Procrastination that allows an abscess to form is to be deplored. Even if the abscess be walled off, its inner wall must be formed by adherent loops of bowel which become much injured by the process of granulation, cicatrization and contraction associated with the healing of the abscess cavity. Intestinal obstruction is not an uncommon result.

